# Molecular Typing and Epidemiology Profiles of Human Adenovirus Infection among Paediatric Patients with Severe Acute Respiratory Infection in China

**DOI:** 10.1371/journal.pone.0123234

**Published:** 2015-04-09

**Authors:** Yamin Li, Weimin Zhou, Yanjie Zhao, Yanqun Wang, Zhengde Xie, Yongliang Lou, Wenjie Tan

**Affiliations:** 1 Key Laboratory of Medical Virology, Ministry of Health, National Institute for Viral Disease Control and Prevention, China CDC, Beijing, China; 2 Institute of Medical Virology, Wenzhou Medical University, Wenzhou, Zhejiang, China; 3 Beijing Pediatric Research Institute, BCH-CMU, Beijing, China; University of Hong Kong, HONG KONG

## Abstract

**Background:**

Human adenoviruses (HAdVs) have been recognised as pathogens that cause a broad spectrum of diseases. The studies on HAdV infection among children with severe acute respiratory infection (SARI) are limited.

**Objective:**

To investigate the prevalence, epidemiology, and genotype of HAdV among children with SARI in China.

**Study Design:**

Nasopharyngeal aspirates (NPAs) or induced sputum (IS) was collected from hospitalised children with SARIs in Beijing (representing Northern China; n = 259) and Zhejiang Province (representing Eastern China; n = 293) from 2007 to 2010. The prevalence of HAdV was screened by polymerase chain reaction (PCR), followed by sequence typing of PCR fragments that targeted the second half of the hexon gene. In addition, co-infection with other human respiratory viruses, related epidemiological profiles and clinical presentations were investigated.

**Results and Conclusions:**

In total, 76 (13.8%) of 552 SARI patients were positive for HAdV, and the infection rates of HAdV in Northern and Eastern China were 20.1% (n = 52) and 8.2% (n = 24), respectively. HAdV co-infection with other respiratory viruses was frequent (infection rates: Northern China, 90.4%; Eastern China, 70.8%). The peak seasons for HAdV-B infection was winter and spring. Additionally, members of multiple species (Human mastadenovirus B, C, D and E) were circulating among paediatric patients with SARI, of which HAdV-B (34/52; 65.4%) and HAdV-C (20/24, 83.3%) were the most predominant in Northern and Eastern China, respectively. These findings provide a benchmark for future epidemiology and prevention strategies for HAdV.

## Introduction

Adenoviruses are ubiquitous, non-enveloped, double-stranded DNA viruses [1]. Human adenoviruses (HAdVs) are classified into 7 species (Human mastadenovirus A to G) and at least 69 recognized genotypes based on serology, whole-genome sequencing, and phylogenetic analyses [1]. The prevalence of different HAdV types varies among different geographical regions [2]. HAdVs have been recognised as pathogens that cause a broad spectrum of diseases [1, 2], including acute respiratory infection (ARI), gastroenteritis, conjunctivitis, cystitis, and meningoencephalitis. ARI is prevalent in children, and is one of the most common causes of morbidity and mortality in the paediatric population in developing countries [3, 4]. Numerous outbreaks of ARI caused by HAdV have been reported during the last decade in many countries including China [5–14]. The HAdV types most commonly found in respiratory samples belong to HAdV-C (HAdV-1, -2, -5, -6) and HAdV-B (HAdV-3, -7) [2, 10–15]; however, severe or even fatal disease outbreaks are predominantly caused by only a few types (such as HAdV-14, -21 and -55) [2, 5–9]. The molecular typing by HAdV hexon sequences can help to accelerate the discrimination of types, resulting in timely epidemiological examinations and improved patient care [16–20]. Several studies have shown the association between severe respiratory infections in adult and HAdV species [7–9]; however, reports among children with severe acute respiratory infection (SARI) in China are limited.

The purpose of this study was to determine the prevalence and genotype (sequencing of the hexon gene after polymerase chain reaction [PCR] screening) of HAdVs among children with SARI in different areas of China from 2007 to 2010. HAdV infections are often associated with the co-infection of bacterial or viral agents [21], frequently leading to severe clinical consequences in hospital patients. Thus, co-infection with other respiratory viruses of HAdV was also investigated.

## Materials and Methods

### Ethical approval

This project was approved by the Institutional Review Boards of the Centre of Disease Control and Prevention of China, the Ethical Review Committee of Beijing Hospital, and the Ethics Committee of Wenzhou Medical College. Individual written informed consent was obtained from the parents or guardians of all of the participants.

### Study population and specimens

We defined a case of SARI according to the World Health Organization case definition for all hospitalised children in whom the onset of illness occurred within 7 days of admission [22]. Basic demographic and clinical information and laboratory results were recorded for each patient using a standardised form. Specimens were collected on the day of admission. Two separate banks of specimens between May 2007 and March 2010 were used in this investigation. The first set consisted of 259 nasopharyngeal aspirate (NPA) specimens collected from paediatric patients with SARI in Beijing (Northern China). The second specimen bank comprised 293 induced sputum (IS) specimens from paediatric patients with SARI in Zhejiang Province (Eastern China). All of the samples were collected from the patients at the first day of admission and placed in viral transport medium and stored at -80°C until tested.

### Laboratory Methods

Nucleic acids (both DNA and RNA) were extracted from 200 μL virus transport medium using the QIAamp MinElute Virus Spin Kit (Qiagen, Hilden, Germany) according to the manufacturer’s instructions.

A partial hexon gene fragment was amplified by PCR to screen and type HAdV infection as previously described [16, 23]. The first-round PCR primers were AdTU7 (5′-GCCACCTTCTTCCCCATGGC-3′) and AdTU4′ (5′- GTAGCGTTGCCGGCCGAGAA-3′) with 1004 bp PCR product; the second-round primers were AdnU-S′ (5′-TTCCCCATGGCCCACAACAC-3′) and AdnU-A (5′-GCCTCGATGACGCCGCGGTG-3′) with 956 bp PCR product. The PCR reactions contained 1.25 U FastStart Taq DNA Polymerase, 10× PCR Buffer with 2 mM MgCl_2_ (Roche Diagnostic Systems Inc., Mannheim, Germany), 0.5 μM of each primer, 200 μM of each deoxynucleoside triphosphate (i.e., dATP, dGTP, dCTP, dTTP; TaKaRa, Shiga, Japan), and 5 μL nucleic acids (first round) or 2 μL first-round PCR product (second round) as a template in a total volume of 25 μL. PCR conditions comprised an initial denaturation step at 94°C for 10 min, followed by 36 cycles of denaturation at 94°C for 1 min, annealing at 50°C for 1 min, and extension at 72°C for 2 min. The final step was an extension at 72°C for 7 min. The second round of PCR was conducted using the same PCR conditions described above for the first round.

After the nested-PCR amplification, 5 μL reaction mixtures was subjected to electrophoresis on a 2% agarose gel containing 0.5 μg ethidium bromide per mL. The positive products were gel purified for DNA sequencing using the QIAquick Gel Extraction Kit (Qiagen) according to the manufacturer’s instructions. DNA sequencing was performed using the Cycle Sequencing Kit (Qiagen) on a DNA analyser.

Specimens positive for HAdV were further tested for co-infection with other respiratory viruses by multiplex PCR and multiple reverse transcription PCR (RT-PCR), including respiratory syncytial virus (RSV), picornaviruses (PIC) including enteroviruses and rhinoviruses, influenza A and B virus, parainfluenza virus (PIV) types 1, 2 and 3, human bocavirus (HBoV), human metapneumovirus (hMPV), and human coronavirus (HCoV), including HCoV-OC43, HCoV-229E, HCoV-NL63, and HCoV-HKU1, as previously described [24, 25].

### Phylogenetic tree and typing

The partial hexon gene sequences have been deposited in GenBank under the accession numbers KM377987-KM378038 and KM877524-KM877547. A total of 28 prototype reference sequences of HAdV used for comparisons with sequences from this study were obtained from GenBank (S1 Table). These were aligned and manually adjusted using Clustal X2 and BioEdit. The phylogenetic tree was drawn based on the hexon region of adenovirus by the neighbour-joining (NJ) method using Molecular Evolutionary Genetics Analysis (MEGA) software version 5.0.

### Statistical analysis

Data were analysed by the chi-squared test using SAS software version 9.2. *P* < 0.05 was considered statistically significant.

## Results

### Demographic and epidemiological data

All of the samples were collected from inpatient children with SARI at hospitals in Beijing (n = 259) and Zhejiang Province (n = 293) from 2007 to 2010. The age and gender distributions are shown in Table 1. The median age of this population is 7 and 7.5 months, respectively. The demographic characteristics of the Northern and Eastern Chinese population were matched well, and no significant differences were observed regarding gender and age distribution (*P* > 0.05).

**Table 1:** Demographic data in this study.

Characteristics		Northern		Eastern		*P* value
		n = 259	%	n = 293	%	
Male/Female		155/104	59.8/40.2	195/98	66.6/33.4	0.1025
Median age		7 months		7.5 months		0.9561
(range)		(1 M to 6 ys 2 Ms)		(0.5 M to 11 ys)		
Age	< 6 months	116	44.8	130	44.4	
	6 months to 1 year	60	23.2	71	24.2	
	1–2 years	43	16.6	51	17.4	
	> 2 years	40	15.4	41	14.0	
HAdV (+)	N = 76	52	20.1	24	8.2	< 0.0001

In total, 76 (13.8%) of 552 SARI patients were positive for HAdV. The prevalence of HAdV among inpatient children with SARI in Northern China was 20.1% (n = 52), which is higher than that in Eastern China (8.2%, n = 24) (*P* < 0.0001). Most of the HAdV infections occurred in children aged 6 months to 2 years (Table 2). The seasonal distribution indicated that most of the HAdV-B infections occurred during the winter and spring (Table 3). However, no season variation was shown for HAdV-C infections in this study.

**Table 2:** Age distribution of HAdV infection.

	< 6 months	6 months to 1 year	1–2 years	> 2 years	*P* value***
	n = 246 (%)	n = 131 (%)	n = 94 (%)	n = 81 (%)	
**Total HAdV**	16 (6.5) *	26 (19.9) * *	**23 (24.5)**	**11 (13.6)**	**< 0.0001**
HAdV-B	9 (3.7)	14 (10.7)	10 (10.6)	4 (4.9)	0.0189
HAdV-C	6 (2.4)	11 (8.4)	13 (13.8)	7 (8.6)	< 0.0001

*, also contain one HAdV-D (HAdV-37)

* *, also contain one HAdV-E (HAdV-4)

*** indicated the statistically difference of the HAdV infection rate among four age groups

**Table 3:** Seasonal distribution of HAdV infection.

	Spring (Mar–May)	Summer (Jun–Aug)	Autumn (Sep–Nov)	Winter (Dec–Feb)	*P* value**
	n = 167 (%)	n = 26 (%)	n = 157 (%)	n = 202 (%)	
**Total HAdV**	33 (19.8) *	**2(7.7)**	**11 (7.0)**	**30 (14.9)**	**0.0057**
HAdV-B	18 (10.8)	0	1 (0.6)	18 (8.9)	< 0.0001
HAdV-C	13 (7.8)	2 (7.7)	10 (6.4)	12 (5.9)	0.8468

*, also contain one HAdV-D (HAdV-37) and one HAdV-E (HAdV-4)

**, indicated the statistically difference of the HAdV infection rate among four seasons

### Phylogenetic analysis and typing of HAdV

**Fig 1:**
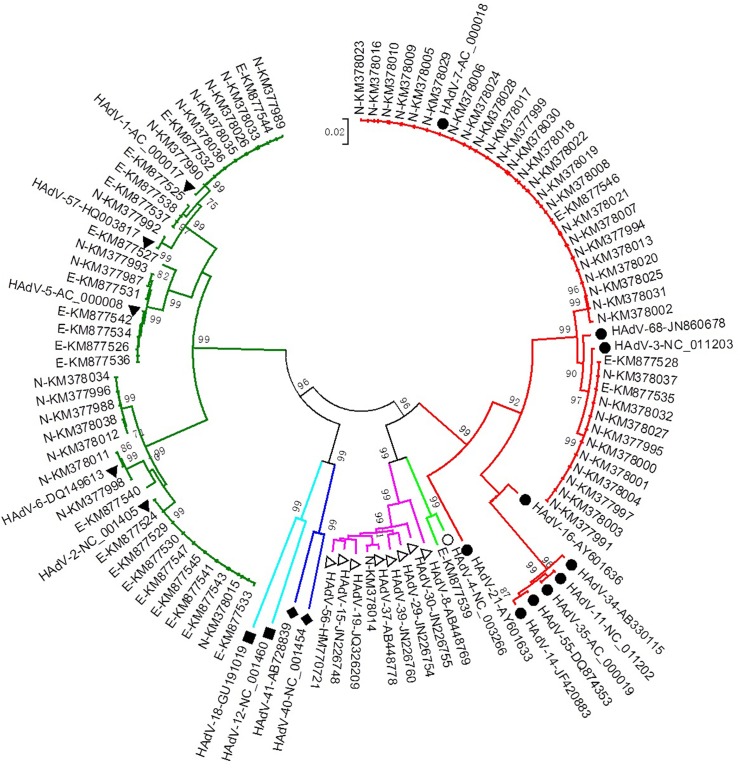
Phylogenetic analysis of HAdV based on the partial hexon gene. The phylogenetic tree was constructed by the neighbour-joining method, and bootstrap values were determined by 1000 replications in MEGA5.0. (Prefix-N: samples from Northern China; Prefix-E: samples from Eastern China; ■, sequences of the reference strains of HAdV-A cut from genomes found in GenBank; ●, sequences of reference strains of HAdV-B; ▼, sequences of reference strains of HAdV-C; Δ, sequences of reference strains of HAdV-D; ○, sequence of reference strain of HAdV-E; ◆, sequences of reference strains of HAdV-F.

The phylogenetic tree was built based on alignment of the nucleotide sequences of the amplicons of HAdV and the relevant prototype sequences from GenBank. Fig 1 and Table 3 show that HAdV-B and HAdV-C were the most prevalent. The strains isolated from Northern China were identified as HAdV-3 (n = 10) and HAdV-7 (n = 24), belonging to HAdV-B; HAdV-1 (n = 6), HAdV-2 (n = 1), HAdV-5 (n = 2), HAdV-6 (n = 2) and HAdV-57 (n = 1), belonging to HAdV-C; and HAdV-37 (n = 1), belonging to HAdV-D(Table 4). The genotypes of most HAdV isolates were identified by alignment to those prototypes from GenBank, with the exception of isolates KM377988, KM377996, KM378012, KM378034, and KM378038, which formed a single separate cluster compared to prototypes HAdV-2 and HAdV-6. HAdV-37 and HAdV-57 were also detected in respiratory specimens, which were reported as the pathogens of keratoconjunctivitis and gastrointestinal, respectively. In contrast, HAdV-C was predominant among the strains from Eastern China with HAdV-1 (n = 5), HAdV-2 (n = 8), HAdV-5 (n = 6), and HAdV-6 (n = 1), whereas HAdV-B also contained several strains from Eastern China with HAdV-3 (n = 2) and HAdV-7 (n = 1), and HAdV-4 (HAdV-E).

**Table 4:** Genotype profiles and co-infection of HAdV.

HAdV	Northern (%)	Eastern (%)	*P* value*
(Total: n = 76)	(n = 52)	(n = 24)	
**HAdV-B**	**34 (65.4)**	**3 (12.5)**	**< 0.0001**
HAdV-3 (n = 12)	10 (19.2)	2 (8.3)	
HAdV-7 (n = 25)	24 (46.2)	1 (4.2)	
**HAdV-C**	**17 (32.7)**	**20 (83.3)**	**< 0.0001**
HAdV-1 (n = 11)	6 (11.5)	5 (20.8)	
HAdV-2 (n = 9)	1 (1.9)	8 (33.3)	
HAdV-5 (n = 8)	2 (3.8)	6 (25.0)	
HAdV-6 (n = 3)	2 (3.8)	1 (4.2)	
HAdV-2/6 (n = 5)	5 (9.6)		
HAdV-57	1 (1.9)		
**HAdV-D**	**1 (1.9)**		
HAdV-37	1 (1.9)		
**HAdV-E**		**1 (4.2)**	
HAdV-4		1 (4.2)	
**HAdV only** (n = 12)	5 (9.6)	7 (29.2)	0.0298
**Co-infection** (n = 64)	47 (90.4)	17 (70.8)	
with RSV (n = 37)	32 (61.5)	5 (20.8)	0.0057
with HRV (n = 25)	22 (42.3)	3 (12.5)	0.0347
with HBoV (n = 22)	14 (26.9)	8 (33.3)	0.1988
with PIV3 (n = 13)	11 (21.2)	2 (8.3)	0.4851
with Flu A (n = 6)	5 (9.6)	1 (4.2)	1.000
with HCoV-OC43 (n = 4)	3 (5.8)	1 (4.2)	1.000

*, indicated the statistically difference of the HAdV infection rate between the Northern and Eastern China.

### Co-infection and clinical profiles

HAdV co-infection with other respiratory viruses among the inpatient children with SARI was found in 47 patients (47/52; 90.4%; 95% confidence interval (CI): 64.5–116.2%) and 17 cases (17/24; 70.8%; 95% CI: 37.2–104.5%) from Northern and Eastern China, respectively, as shown in Table 4. The dominant co-infection with HAdV was RSV, human rhinovirus (HRV), HBoV, and PIV3. Single infection was found in 12 (15.8%) from 76 HAdV positive samples in this study. Demographic, typing and clinical profiles with single HAdV infection are shown in Table 5, which indicated that dominant types for single HAdV infection was HAdV-7 (3 cases), HAdV-1 (3 cases) and HAdV-2 (2 cases).

**Table 5:** Demographic, typing and clinical profiles with single HAdV infection cases in this study.

Area & Sample No.	Gender	Age	Sampling time	Clinical manifestation	Clinical diagnosis	HAdV typing
**Northern**						
168	F	1Y6M	2009-02-18	Fever, cough, expectoration	Pneumonia	HAdV-7
235	M	4Y	2009-03-31	Fever, cough, expectoration, pharyngalgia, runny nose	Pneumonia	HAdV-7
289	F	2Y3M	2009-06-04	Fever, cough, vomiting	Pneumonia	HAdV-3
347	F	4Y6M	2009-11-11	Fever, cough,	Pneumonia	HAdV-2/6
350	M	4Y2M	2009-11-19	Fever, cough, diarrhea	Pneumonia	HAdV-1
**Eastern**						
103	F	0.6M	2007-10-31	Fever, cough,	Pneumonia	HAdV-2
212	M	1Y	2008-02-13	Fever, cough, expectoration, gastrointestinal symptom	Bronchitis	HAdV-1
217	M	9M	2008-02-12	Fever, cough,	Pneumonia	HAdV-1
244	F	2Y	2008-03-06	Fever, cough, nasal obstruction, runny nose	Pneumonia	HAdV-2
256	M	8M	2008-03-10	Cough, expectoration, diarrhea	Bronchitis, enteritis	HAdV-5
301	M	1Y1M	2008-04-10	Fever, cough, chills	Pneumonia	HAdV-2
302	F	4Y	2008-04-12	Fever, cough, expectoration	Bronchitis	HAdV-7

Based on the analysis of clinical records, the clinical manifestation of the patients with HAdV infection included fever, cough, and sputum. A few cases showed signs of a runny nose, nasal congestion, and gastrointestinal symptoms, such as diarrhoea and vomiting (Table 5). Because the prevalence of co-infection of HAdV with other viruses is higher than that of HAdV infection alone, it is difficult to associate the clinical profiles with HAdV infection or specific types.

## Discussion

Among the seven recognised HAdV species (HAdV-A to G), HAdV-B (particularly HAdV-3, HAdV-7, HAdV-11, HAdV-14, HAdV-21) and E (HAdV-4) have more often been associated with epidemic ARI [4–6, 12–15, 19]. Rapid detection of these viruses would enhance the outbreak response. We reported the prevalence of multiple HAdV types (HAdV-B, HAdV-C, HAdV-D HAdV-E) in paediatric patients with SARI in China from 2007 to 2010 (overlapping the H1N1 pandemic period). Consistent with previous reports [6, 11–15], the HAdV-B and HAdV-C were the most common serotypes for ARI, and co-infection with other respiratory viruses was frequent (64 from 76, especially co-infection with RSV, HRV, HBoV and PIV3). And 32 from 76 were co-infected by two or more viruses. The previous studies on co-infection with other respiratory viruses of HAdV were limited. Our data showed the higher number of co-infection might be resulted from samples (from the severe paediatric patients) and method (molecular detection for multiple viruses, optimized SOP….). Because of the high co-infection rate, the specific clinical manifestations associated with HAdV infection are not clear and its etiological significance in our study was uncertain.

The severity of infection associated with HAdV varies depending on the different HAdV types [10, 15]. In addition, the prevalence of different HAdV serotypes varies among different geographical regions [12–15]. Our study indicated that HAdV was frequently detected in SARI children from 2008 to 2010 (52/259, 20.1%) in Northern China; this frequency was higher than that of cases in Eastern China (24/293, 8.2%) and in other studies [10–12, 19]. The reasons for the marked differences are unknown. We suggest that the cause may be the difference in species of the sample as well as the disease severity in the population studied. However, further investigation is needed. Furthermore, among the 76 HAdV samples, 25 (32.9%) were identified as HAdV-7 (HAdV-B), and 15 (19.7%) as HAdV-3 (HAdV-B), representing the most common molecular types. Previous studies have found that HAdV-B was the most common species in southern China [26], and HAdV-3 was the predominant type of HAdV-B. And another study reported HAdV-3 (HAdV-B) outbreak associated with febrile respiratory disease in Eastern China in 2011[27]. In the present study the most dominant HAdV-C strains were HAdV-1 (11, 14.47%), followed by HAdV-2 (9, 11.84%), and HAdV-5 (8, 10.52%). Other HAdV-C types including HAdV-6, HAdV-2/6 and HAdV-57, as well as HAdV-D (HAdV-37) and HAdV-E (HAdV-4) types were also observed.

Recombination is a well-known feature of adenovirus genetics [28, 29]. It typically occurs only between strains of the same species [29]; interspecies recombinants are uncommon [20]. It is noteworthy that we found a peculiar HAdV-2/6 cluster in the present study, likely the recombinants of HAdV-2 and HAdV-6. We have amplified the full hexon from one of HAdV-2/6 recombinants (GenBank accession No. KP696777). Its position on the phylogenetic tree indicated that the intertypic (HAdV-2/6) recombination occurred in the second half of the hexon gene (data not shown). However, confirmation of this event requires the sequencing of the complete genome.

Previous reports have indicated that HAdV-37 was associated with epidemic keratoconjunctivitis [30, 31], and HAdV-57 was isolated from stool specimens of healthy or gastroenteritis-affected children [4, 32]. Surprisingly, we found HAdV-37 (KM378014) and HAdV-57 (KM377992) each in single respiratory samples of inpatient children with SARI, suggesting that they may not be limited to the conjunctiva and the gastrointestinal tract.

In conclusion, the present study reported the circulating HAdV types and their prevalence in paediatric patients with SARI in China. HAdV was frequently (76/552, 13.77%) detected, and HAdV-B and HAdV-C were the most predominant in Northern and Eastern China, respectively. However, we only amplified relatively conserved partial hexon sequences rather than the hypervariable region (HVR), perhaps resulting in the loss of the recombinant and variant information. More extensive studies will be needed to address the prevalence and geographic distribution of dominant genotypes of HAdV using well-matched control groups and sequential samples collected over a longer time. Finally, to the best of our knowledge, HAdV-37 (KM378014) and HAdV-57 (KM377992) are the first strains of these genotypes to be reported in inpatient children with SARI.

## Supporting Information

S1 Table Reference strain used for phylogenic analysis. (DOCX)
