# Supplementary material for: Molecular Typing and Epidemiology Profiles of Human Adenovirus Infection among Paediatric Patients with Severe Acute Respiratory Infection in China
Source: PLoS One. 2015 Apr 9;10(4):e0123234. doi: 10.1371/journal.pone.0123234 (PMC4391708; doi:10.1371/journal.pone.0123234)
Supplement: S1 Table — (DOCX) [file pone.0123234.s001.docx]

**Supplement** Table 1 Reference strain used for phylogenic analysis

| GenBank no. | Genotype | Species | Nation |
| --- | --- | --- | --- |
| NC_001460 | HAdV-12 | A | USA |
| GU191019 | HAdV-18 | A | USA |
| NC_011203 | HAdV-3 | B | USA |
| AC_000018 | HAdV-7 | B | USA |
| JF420883 | HAdV-14 | B | China |
| AY601636 | HAdV-16 | B | USA |
| AY601633 | HAdV-21 | B | USA |
| AB330115 | HAdV-34 | B | Japan |
| AC _000019 | HAdV-35 | B | USA |
| DQ874353 | HAdV-55 | B | China |
| JN860678 | HAdV-68 | B | USA |
| AC_000017 | HAdV-1 | C | USA |
| NC_001405 | HAdV-2 | C | USA |
| AC_000008 | HAdV-5 | C | USA |
| DQ149613 | HAdV-6 | C | Austria |
| KF835458 | HAdV-57 | C | China |
| AB448769 | HAdV-8 | D | Japan |
| JN226748 | HAdV-15 | D | USA |
| JQ326209 | HAdV-19 | D | USA |
| JN226754 | HAdV-29 | D | USA |
| JN226755 | HAdV-30 | D | USA |
| AB448778 | HAdV-37 | D | Japan |
| JN226760 | HAdV-39 | D | USA |
| HM770721 | HAdV-56 | D | USA |
| NC_003266 | HAdV-4 | E | U.K. |
| NC_001454 | HAdV-40 | F | USA |
| AB728839 | HAdV-41 | F | Japan |
